# Noninvasive positive-pressure ventilation for children with acute asthma: a meta-analysis of randomized controlled trials

**DOI:** 10.3389/fped.2023.1167506

**Published:** 2023-04-28

**Authors:** Jiajia Dai, Libo Wang, Fang Wang, Lu Wang, Qingfen Wen

**Affiliations:** ^1^ Department of Respiratory Medicine, National Children's Medical Center, Children’s Hospital of Fudan University, Shanghai, China; ^2^ Department of Pediatrics, Jinshan Hospital, Fudan University, Shanghai, China

**Keywords:** asthma, children, noninvasive positive-pressure 5entilation, efficacy, meta-analysis

## Abstract

**Background:**

Noninvasive positive-pressure ventilation (NPPV) can be effective in children with acute asthma. However, clinical evidence remains limited. The objective of the meta-analysis was to systematically assess NPPV's effectiveness and safety in treating children with acute asthma.

**Methods:**

Relevant randomized controlled trials were obtained from electronic resources, including PubMed, Embase, Cochrane's Library, Wanfang, and CNKI databases. The influence of potential heterogeneity was taken into account before using a random-effect model to pool the results.

**Results:**

A total of 10 RCTs involving 558 children with acute asthma were included in the meta-analysis. Compared to conventional treatment alone, additional use of NPPV significantly improved early blood gas parameters such as the oxygen saturation (mean difference [MD]: 4.28%, 95% confidence interval [CI]: 1.51 to 7.04, *p *= 0.002; *I*^2^ = 80%), partial pressure of oxygen (MD: 10.61 mmHg, 95% CI: 6.06 to 15.16, *p *< 0.001; *I*^2^ = 89%), and partial pressure of carbon dioxide (MD: −6.29 mmHg, 95% CI: −9.81 to −2.77, *p *< 0.001; *I*^2^ = 85%) in the arterial blood. Moreover, NPPV was also associated with early reduced respiratory rate (MD: −12.90, 95% CI: −22.21 to −3.60, *p *= 0.007; *I*^2 ^= 71%), improved symptom score (SMD: −1.85, 95% CI: −3.65 to −0.07, *p *= 0.04; *I*^2^ = 92%), and shortened hospital stay (MD: −1.82 days, 95% CI: −2.32 to −1.31, *p *< 0.001; *I*^2 ^= 0%). No severe adverse events related to NPPV were reported.

**Conclusions:**

NPPV in children with acute asthma is associated with improved gas exchange, decreased respiratory rates, a lower symptom score, and a shorter hospital stay. These results suggest that NPPV may be as effective and safe as conventional treatment for pediatric patients with acute asthma.

## Introduction

As a prevalent chronic disease in childhood, asthma exacerbations or acute attacks have become one of the most common reasons for emergency department visits or hospitalization (1, 2). Although severe acute asthma can be prevented, it is a leading cause of pediatric patient mortality, especially in patients from developing nations (3, 4). An acute asthma attack refers to progressive respiratory symptoms (wheezing, cough, or dyspnea) and impaired pulmonary function (3, 4). Pathophysiologically, asthma exacerbation is characterized by bronchospasm, inflamed airways, mucous plugging, and an imbalance between ventilation and perfusion (5). Accordingly, conventional therapy for acute severe asthma includes the rapid reversal of airway obstruction and oxygen supplementation to correct hypoxia and prevent relapse (5). Despite these efforts, some children with acute severe asthma continue to have breathless symptoms, and respiratory failure may develop in these patients (6). For these children, noninvasive positive-pressure ventilation (NPPV) has been suggested to be an additional efficacious treatment (7). Early use of NPPV in high-risk children with acute severe asthma could theoretically reduce the work of breathing and give more time for the pharmacological treatment to achieve maximal efficacy, thereby reducing the risk of respiratory failure and subsequent need for mechanical ventilation (8). Although experimental clinical observations support the use of NPPV for children with acute severe asthma, evidence based on clinical trials is still lacking (9–11). In this meta-analysis, we aimed to systematically evaluate the efficacy and safety of NPPV for children with acute asthma by summarizing the results of available randomized controlled trials (RCTs).

## Methods

During its design and implementation, this study followed PRISMA (Preferred Reporting Items for Systematic Reviews and Meta-Analyses) (12, 13) and Cochrane Handbook (14) guidelines.

### Search strategy

The following strategies were used to search the databases PubMed, Embase, Cochrane Library, Wanfang, and China National Knowledge Infrastructure (CNKI): (1) “non-invasive” OR “noninvasive” OR “positive-pressure” OR “positive pressure” OR “pressure support” OR “pressure-support” OR “positive airway” or “positive-airway” OR “pressure control” OR “pressure-control” OR “bi-level” OR “NPPV” OR “NIPPV” OR “CPAP” OR “BiPAP” OR “ventilation” OR “ventilating”; (2) “asthma” OR “wheeze” OR “wheezing”; (3) “child” OR “children” OR “adolescent” OR “pediatric” OR “paediatric” OR “infant” OR “neonate” OR “newborn” OR “toddler”; and (4) “random” OR “randomly” OR “randomized” OR “randomised” OR “placebo”. Only studies involving human participants and published as full-length articles in a journal subject to peer review were considered. In addition to the final database search, references to relevant reviews and original articles were also investigated. The last database search was conducted on 31 December 2022.

### Study selection

The PICOS principle was followed in designating the meta-analysis inclusion criteria.

P (patients): Children (<18 years) with a confirmed diagnosis of acute asthma.

I (intervention): A treatment group of NPPV based on conventional therapy.

C (control): A control group of conventional therapy alone.

O (outcomes): Between-group difference of changes of one or more of the following results: (1) acute changes of parameters of blood gas analysis (BGA) evaluated within four hours after NPPV treatment, including oxygen saturation (SaO_2_), partial pressure of oxygen (PaO_2_), and partial pressure of carbon dioxide (PaCO_2_) in the arterial blood; (2) acute changes of the respiratory rate (RR) and/or clinical symptoms scores of asthma [such as the Clinical Asthma Score (CAS)] within four hours after NPPV treatment; and (3) length of hospitalization (LOH).

S (study design): Parallel-group or crossover RCTs published as full-length papers in Chinese or English.

Non-randomized studies, studies enrolling adult patients, studies with pediatric patients of other respiratory diseases rather than acute asthma, studies without the intervention of NPPV, or studies not reporting relevant results were excluded. Studies with a high-flow nasal cannula (HFNC) were also excluded because HFNC is not a form of NPPV. The key difference between HFNC and NPPV is that compared to HFNC, NPPV can create a much higher gas flow rate and positive airway pressure (15). In clinical practice, HFNC is used as a midway point between low-flow oxygen devices and NPPV (15). The study with the largest sample size was included in the meta-analysis for studies with overlapping patient populations.

### Data collection and quality evaluation

Two authors worked independently on database searches, data collection, and quality assessment. If there were any disagreements, they were discussed with the corresponding author. We gathered information about each study's first author, publication year, study country, study design (blind or open-label), patient information (diagnosis, number of patients, and mean age), clinical setting, details about NPPV treatment and controls, treatment duration, and outcomes. The Cochrane Risk of Bias Tool was used to assess the quality of the included RCTs (14) by assigning random sequences, concealing allocations, blinding participants and personnel, blinding outcomes assessors, incomplete outcomes data, and selective outcome reporting.

### Statistical analysis

The mean difference (MD) with a 95% confidence interval represented the effects of NPPV on the BGA parameters, RR, and LOH (CI). Because different scores were used, the effects of NPPV on clinical symptom scores were presented as standardized mean difference (SMD) and 95% CI. The Cochrane's Q test was used to evaluate heterogeneity (14). The *I*^2^ statistic was also calculated, and an *I*^2 ^> 50% denotes significant heterogeneity(16). A random-effects model was used when calculating pooled analyses because it considers potential heterogeneity and yields more generalized results (14). In order to assess how each study affected the combined results, influencing analyses were carried out by removing one study at a time from the meta-analysis (14). Visual examination of funnel plots and the Egger's regression asymmetry test were used to assess publication bias (17). According to the guidelines in Cochrane's Handbook, shared intervention groups in studies with multiple comparisons were equally split and included as independent comparisons to avoid a unit-of-analysis error (14). Differences for *p *< 0.05 were deemed statistically significant. The RevMan (Version 5.1; Cochrane, Oxford, UK) and Stata (Version 12.0; Stata Corporation, College Station, TX) software packages were used to conduct the statistical analyses.

## Results

### Literature search

**Figure 1:**
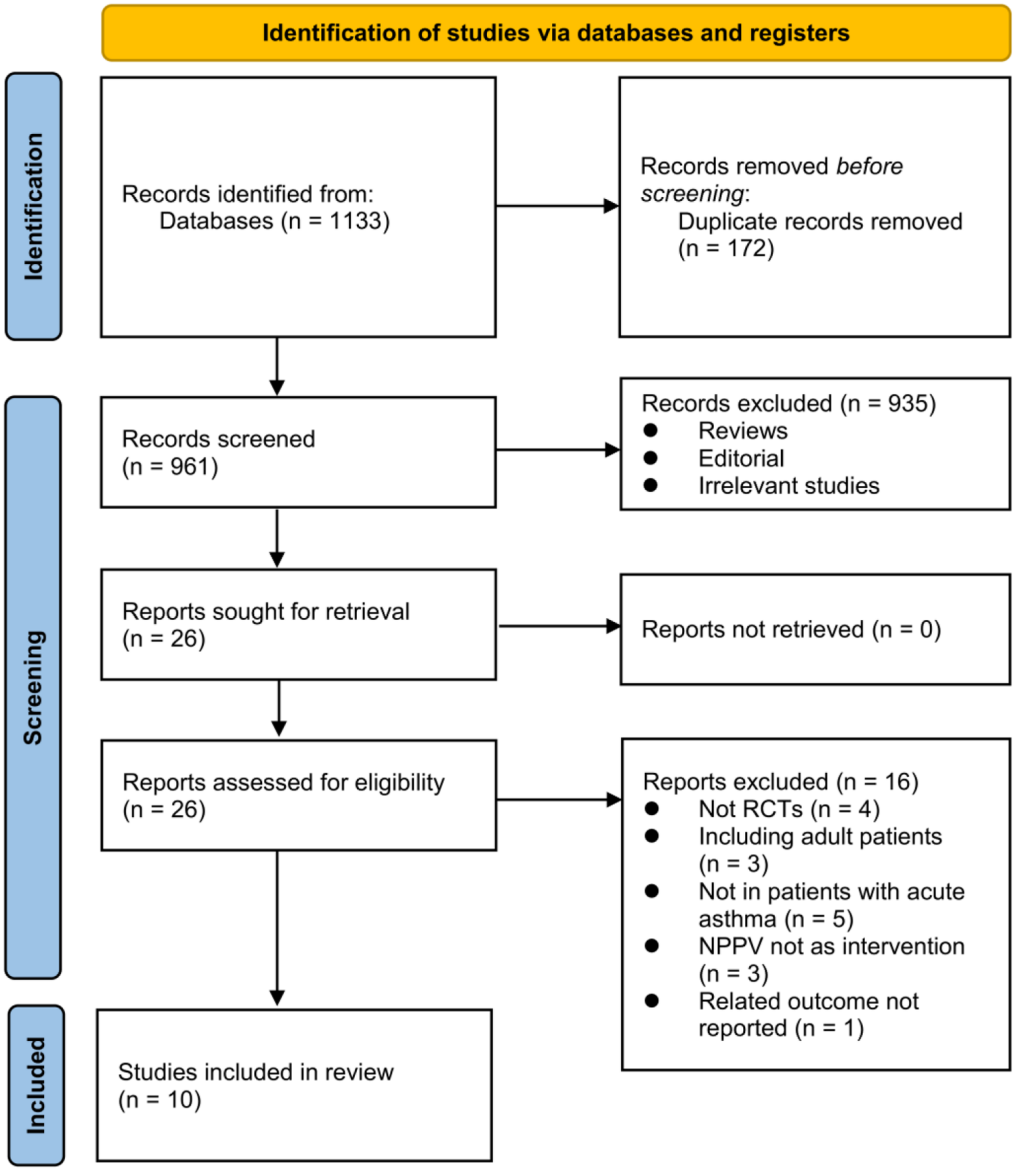
Flowchart of literature search.

The study flow chart is shown in Figure 1. In a nutshell, database searches turned up 1,133 articles, and 961 of them were found after duplicate records were eliminated. Following that, 935 articles were disqualified based on their titles and abstracts, mainly because they had nothing to do with the objective of the meta-analysis. A total of 16 of the 26 articles that had received full-text reviews were later eliminated for the reasons shown in Figure 1. Ten RCTs (17–26) were ultimately determined to be qualified for the meta-analysis.

### Study characteristics and data quality

Tables 1, 2 provide a summary of the included studies. Overall, ten RCTs (18–27) involving 558 children with acute asthma were included in the meta-analysis. These studies were conducted in the United States and China and published between 2004 and 2021. As for the study design, all included studies were parallel-group RCTs except one crossover RCT (18). Two studies were performed in a pediatric intensive care unit (PICU) (18, 22) and one in the emergency department (25). The remaining studies were in pediatric wards (19–21, 23, 24, 26, 27). The sample sizes of the included studies were limited, varying from 20 to 94. The mean ages of the children were between 3.2 and 8.6 years, and the proportions of males were 51% to 72%. As for the comorbidities, three studies excluded patients with children with other pulmonary diseases, congenital heart diseases, or heart failure (23, 25, 27). The conventional therapies included inhaled beta 2-agonists and intravenous corticosteroids, which were balanced between the intervention and control groups, as mentioned in the original studies. Besides conventional therapy, NPPV was applied additionally in patients of the intervention group, while patients from the control groups received conventional therapy alone (including oxygen supplementation). As for the models of NPPV, bi-level positive airway pressure (BiPAP) was used in seven studies (18–20, 22–24, 26), and other models such as pressure support ventilation (PSV) and positive end-expiratory pressure (PEEP), and positive expiratory pressure (PEP) were used in the other studies (21, 25, 27). By definition, BiPAP delivers a set positive airway pressure during expiration and a higher pressure during inspiration to support inspiratory effort (28); PSV is a mode of positive pressure mechanical ventilation in which the patient triggers every breath, which could be used with PEEP (29); PEEP refers to the positive pressure that will remain in the airways at the end of the respiratory cycle (end of exhalation) that is greater than the atmospheric pressure in mechanically ventilated patients (30); PEP provides a back pressure to the airways during expiration, which is expected to improve respiratory status in acute asthma by recruiting collapsed alveoli, reversing atelectasis, and improving ventilation-perfusion mismatch (25). The observation duration was within PICU in one study (18) and within hospitalization in the others (19–27). Using Cochrane's Risk of Bias Tool, Table 3 provides a detailed analysis of the included RCTs. One included study was double-blind (25), while the others were open-label (18–24, 26, 27). Three studies reported random sequence generation details (19, 21, 27), and the details of allocation concealment were not reported. No evidence of incomplete outcome data, selective reporting, or other sources of bias was detected for all of the included RCTs.

**Table 1:** Characteristics of the included patients.

Study	Country	Design	Diagnosis	Setting	Patient number	Age (years)	Male (%)	Severity score	Comorbidities
Thill 2004	USA	R, OL, CO	Children with increased work of breathing, wheezing, and dyspnea, and a CAS > 3	PICU	20	Median: 4	NR	CAS: 3–8	NR
Yuan 2011	China	R, OL	Children with acute severe asthma	Pediatric ward	40	Mean: 7.9 ± 1.1	60	NR	HF: 17.5%
He 2011	China	R, OL	Children with acute severe asthma	Pediatric ward	57	Mean: 5.6 ± 1.9	56.1	NR	NR
Chen 2011	China	R, OL	Children with acute severe asthma	Pediatric ward	60	Mean: 8.6 ± 2.3	60	NR	HF excluded
Basnet 2012	USA	R, OL	Children with acute severe asthma	PICU	20	Median: 4	55	CAS: 3–8	NR
Liu 2012	China	R, OL	Children with acute severe asthma	Pediatric ward	94	Mean: 3.2 ± 1.1	62.8	NR	Children with other pulmonary diseases, CHD, or HF excluded
Yang 2014	China	R, OL	Children with acute severe asthma	Pediatric ward	52	NR	NR	NR	NR
Navananda 2017	USA	R, DB	Children with acute asthma exacerbations	ED	52	Mean: 7.8 ± 4.2	71.2	PAS: 7–12	Children with other pulmonary diseases, CHD, or HF excluded
Gao 2018	China	R, OL	Children with acute severe asthma	Pediatric ward	80	Mean: 5.7 ± 2.1	51.3	NR	NR
Jiang 2021	China	R, OL	Children with acute severe asthma	Pediatric ward	83	Mean: 3.2 ± 1.5	53	NR	Children with other pulmonary diseases, CHD, or HF excluded

PICU, pediatric intensive care unit; R, randomized; OL, open label; DB, double blind; CO, crossover; NR, not reported; ED, emergency department; CAS, clinical asthma score; PAS, pulmonary asthma score; HF, heart failure; CHD, congenital heart disease.

**Table 2:** Characteristics of the intervention and outcomes reported.

Study	Background treatment	Intervention mode and ventilatory settings	Control	Observation duration	Outcomes reported
Thill 2004	Inhaled beta 2-agonists (continuous nebulized albuterol, 10 mg/h), and intravenous corticosteroids (methylprednisolone, 1–2 mg/kg)	BiPAP, S/T model, with a tight-fitting, nasal mask, with an IPAP of 10 cm H_2_O and an EPAP of 5 cm H_2_O, with humidification, nebulized beta 2-agonists, and supplemental oxygen administered	High-flow oxygen supplementation	During ICU stay	RR, and CAS
Yuan 2011	Anti-bronchospasm (including inhaled beta 2-agonists and intravenous corticosteroids), expectorant, correction of acid-base, water-electrolyte balance, and treatments for HF if necessary	PSV + PEEP, SIMV model, nasal mask, PSV: 10–20 cm H_2_O, PEEP: 3–5 cm H_2_O	Nasal oxygen supplementation	During hospitalization	SaO_2_, PaO_2_, PaCO_2_
He 2011	Inhaled beta 2-agonists and intravenous corticosteroids	BiPAP, S/T model, with a tight-fitting nasal mask, with an IPAP of 5–10 cm H_2_O and an EPAP of 2–3 cm H_2_O	Nasal oxygen supplementation	During hospitalization	LOH
Chen 2011	Anti-bronchospasm (including inhaled beta 2-agonists and intravenous corticosteroids), expectorant, correction of acid-base, water-electrolyte balance	BiPAP, S/T model, with a tight-fitting nasal mask, with an IPAP of 5–10 cm H_2_O and an EPAP of 3–5 cm H_2_O	Nasal oxygen supplementation	During hospitalization	SaO_2_, PaO_2_, PaCO_2_, and LOH
Basnet 2012	Continuous nebulized albuterol (0.5 mg/kg; maximum, 15 mg/kg), intravenous methylprednisolone 2 mg/kg/day (maximum, 80 mg/day). Magnesium sulfate and helium-oxygen (heliox) mixture added at the attending physician's discretion	BiPAP, masks with gel seals, with an IPAP of 8 cm H_2_O and an EPAP of 5 cm H_2_O, humidification, albuterol, and supplemental oxygen administered	Oxygen supplementation	During hospitalization	RR, CAS, and LOH
Liu 2012	Inhaled beta 2-agonists and intravenous corticosteroids	BiPAP, S/T model, with a tight-fitting nasal mask, with an IPAP of 5–10 cm H_2_O and an EPAP of 2–5 cm H_2_O	Oxygen supplementation	During hospitalization	SaO_2_, PaO_2_, PaCO_2_
Yang 2014	Anti-bronchospasm (including inhaled beta 2-agonists and intravenous corticosteroids), expectorant, correction of acid-base, water-electrolyte balance	BiPAP, S/T model, with a tight-fitting nasal mask, with an IPAP of 5–10 cm H_2_O and an EPAP of 2–3 cm H_2_O	Oxygen supplementation	During hospitalization	LOH
Navananda 2017	Combined nebulized ipratropium bromide 0.5 mg and albuterol (2.5 mg for less than 20 kg, 5 mg for 20 kg or more) for a total of 3 doses, and systemic steroids (prednisone 2 mg/kg to a maximum of 60 mg or dexamethasone 0.6 mg/kg to a maximum of 16 mg)	PEP, flow rate (start at 5 L/min), airway pressure (10–20 cm H_2_O), and number (4 cycles) and duration of cycles (12 breaths per cycle)	Oxygen supplementation	During hospitalization	PAS
Gao 2018	Anti-bronchospasm (including inhaled beta 2-agonists and intravenous corticosteroids), expectorant, correction of acid-base, water-electrolyte balance	BiPAP, S/T model, with a tight-fitting nasal mask, with an IPAP of 12–15 cm H_2_O and an EPAP of 2–5 cm H_2_O	Oxygen supplementation	During hospitalization	PaO_2_, PaCO_2_
Jiang 2021	Anti-bronchospasm (including inhaled beta 2-agonists and intravenous corticosteroids), expectorant, correction of acid-base, water-electrolyte balance	PEEP, S/T model, with a tight-fitting nasal mask, with EPAP of 3–5 cm H_2_O	Oxygen supplementation	During hospitalization	SaO_2_, PaO_2_, PaCO_2_

S/T, spontaneous/timed, IPAP, inspiratory positive airway pressure; EPAP, expiratory positive airway pressure; SIMV, synchronized intermittent mandatory ventilation; BiPAP, bi-level positive airway pressure; PSV, pressure support ventilation; PEEP, positive end-expiratory pressure; PEP, positive expiratory pressure; LOH, length of hospitalization; SaO_2_, oxygen saturation in the arterial blood; PaO_2_, the partial pressure of oxygen in the arterial blood; PaCO_2_, partial pressure of carbon dioxide in the arterial blood.

**Table 3:** Study quality evaluation via cochrane's risk of bias tool.

Study	Random sequence generation	Allocation concealment	Blinding of participants	Blinding of outcome assessment	Incomplete outcome data addressed	Selective reporting	Other sources of bias
Thill 2004	Unclear	Unclear	High risk	High risk	Low risk	Low risk	Low risk
Yuan 2011	Low risk	Unclear	High risk	High risk	Low risk	Low risk	Low risk
He 2011	Unclear	Unclear	High risk	High risk	Low risk	Low risk	Low risk
Chen 2011	Low risk	Unclear	High risk	High risk	Low risk	Low risk	Low risk
Basnet 2012	Unclear	Unclear	High risk	High risk	Low risk	Low risk	Low risk
Liu 2012	Unclear	Unclear	High risk	High risk	Low risk	Low risk	Low risk
Navananda 2017	Unclear	Unclear	Low risk	Low risk	Low risk	Low risk	Low risk
Gao 2018	Unclear	Unclear	High risk	High risk	Low risk	Low risk	Low risk
Jiang 2021	Low risk	Unclear	High risk	High risk	Low risk	Low risk	Low risk

### Efficacy outcomes: acute changes of BGA parameters

**Figure 2:**
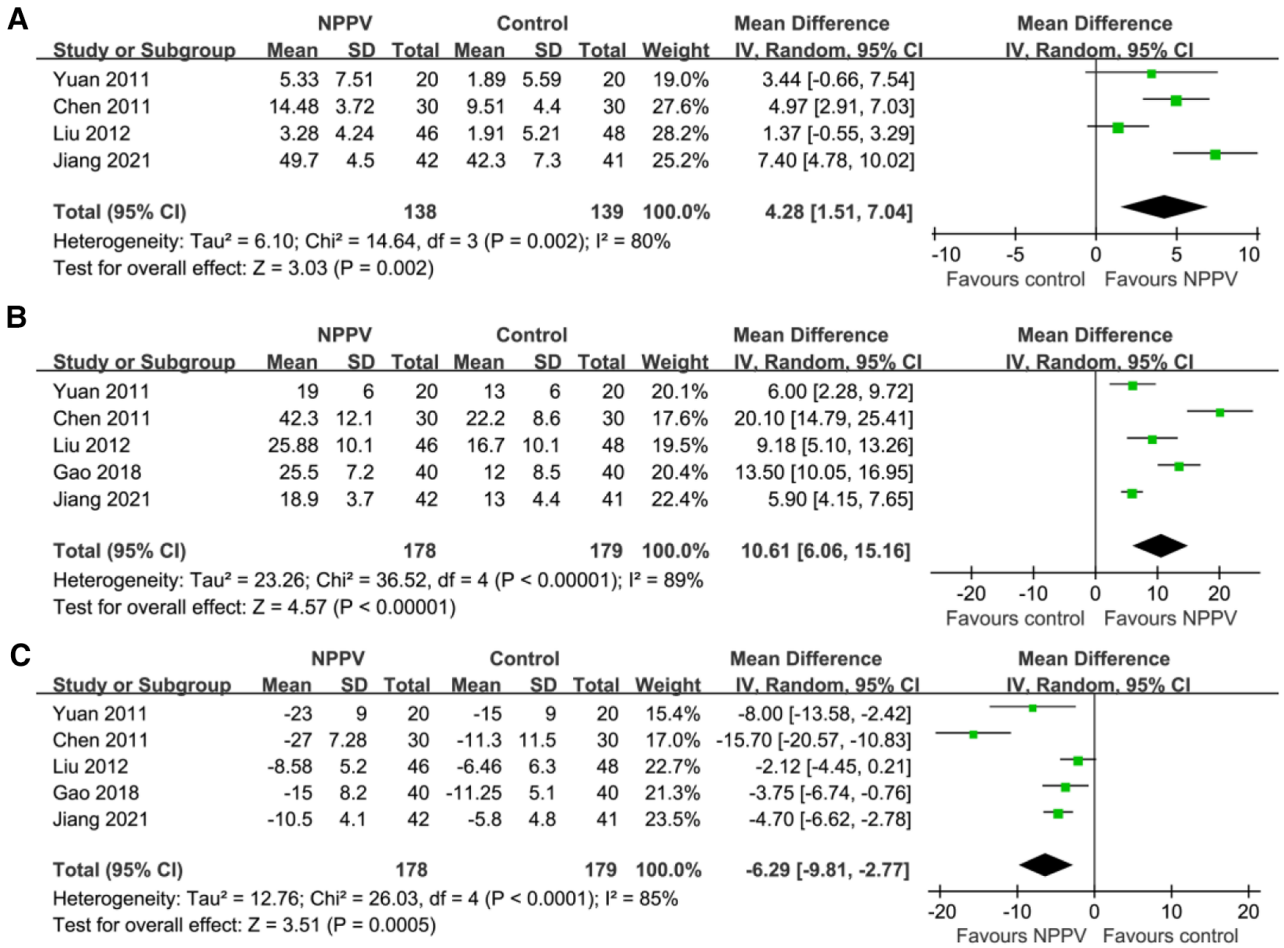
Forest plots for the meta-analysis evaluating the influences of NPPV on acute changes of BGA parameters in children with acute asthma; (**A**) Meta-analysis for the influences of NPPV on SaO_2_; (**B**) Meta-analysis for the influences of NPPV on PaO_2_; and (**C**) meta-analysis for the influences of NPPV on PaCO_2_.

Pooling the results of four studies (19, 21, 23, 27) including 277 patients showed that compared to conventional treatment alone, additional use of NPPV significantly improved SaO_2_ (MD: 4.28%, 95% CI: 1.51 to 7.04, *p *= 0.002; *I*^2^ = 80%; Figure 2A) within four hours after the initiation of the therapy. In addition, pooled results of five studies (19, 21, 23, 26, 27) including 357 patients indicated that NPPV also improved PaO_2_ (MD: 10.61 mmHg, 95% CI: 6.06 to 15.16, *p *< 0.001; *I*^2^ = 89%; Figure 2B) and PaCO_2_ (MD: −6.29 mmHg, 95% CI: −9.81 to −2.77, *p *< 0.001; *I*^2^ = 85%; Figure 2C) acutely compared to controls with conventional treatment only. The results were not affected by excluding one study at a time from the analysis.

### Efficacy outcomes: acute changes of RR and symptom scores

**Figure 3:**
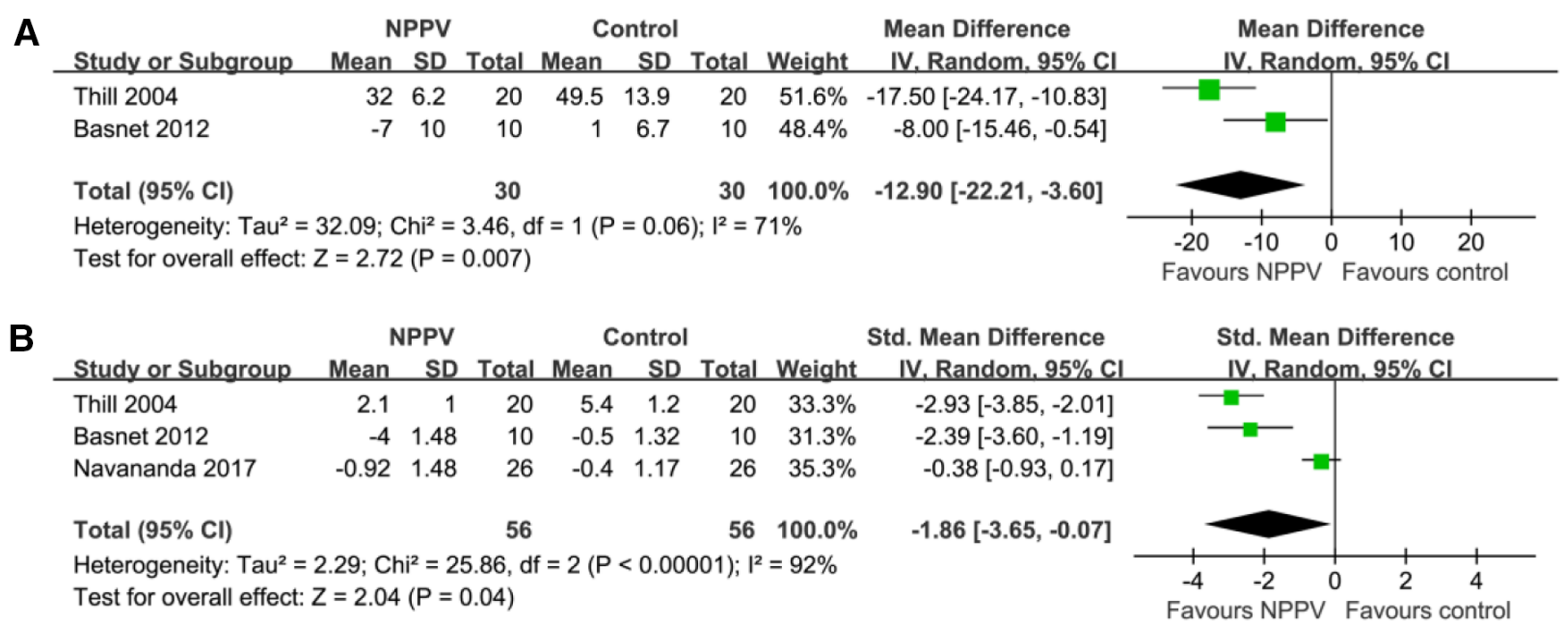
Forest plots for the meta-analysis evaluating the influences of NPPV on acute changes of RR and symptoms score in children with acute asthma; (**A**) Meta-analysis for the influences of NPPV on acute change of RR; and (**B**) meta-analysis for the influences of NPPV on acute change of symptoms score.

Pooled results of two studies (18, 22) including 60 patients suggested that NPPV was also associated with the early reduced respiratory rate (MD: −12.90, 95% CI: −22.21 to −3.60, *p *= 0.007; *I*^2 ^= 71%; Figure 3A). Besides, three studies (18, 22, 25) reported a change in symptom scores following treatment. Among them, the clinical asthma score (CAS) was reported in two studies (18, 22), which was a symptom-based severity score of acute asthma incorporating three domains, such as increased work of breathing, wheezing, and dyspnea (31). In another study (25), the pulmonary asthma score (PAS), which is a pediatric asthma severity scoring system, includes measures of respiratory rate, oxygen saturation, auscultatory findings, retractions, and symptoms of dyspnea (32). Subsequently, pooling the results of three studies (18, 22, 25) including 112 patients demonstrated that NPPV could significantly improve the symptom score (SMD: −1.85, 95% CI: −3.65 to −0.07, *p *= 0.04; *I*^2^ = 92%; Figure 3B) within four hours after the initiation of the treatment for children with acute asthma compared to controls with conventional treatment alone.

### Efficacy outcomes: LOH

**Figure 4:**
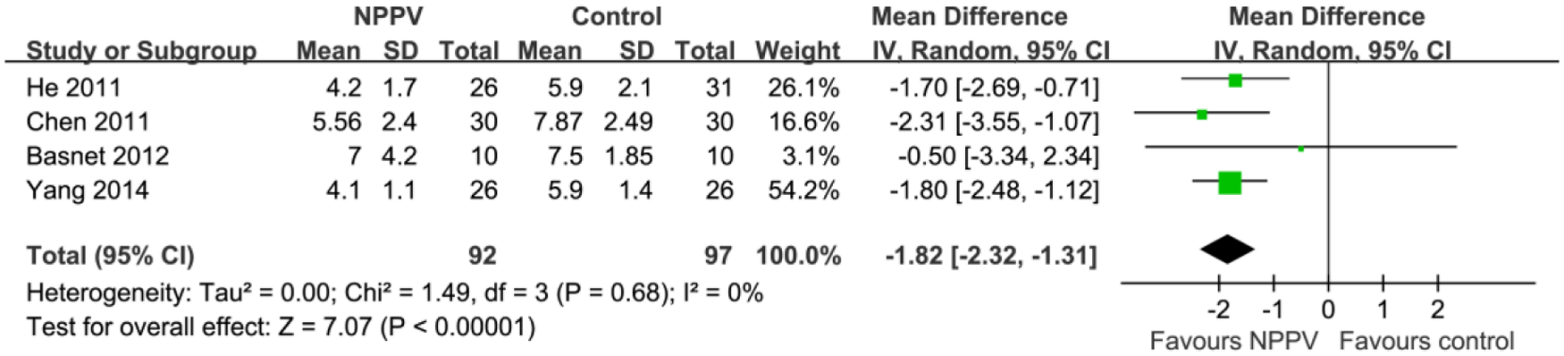
Forest plots for the meta-analysis evaluating the influences of NPPV on LOH.

Four studies including 189 patients reported the outcome of LOH (19, 20, 22, 24). The meta-analysis showed that LOH was significantly decreased for pediatric patients with acute asthma who received NPPV compared to controls with conventional treatment alone (MD: −1.82 days, 95% CI: −2.32 to −1.31, *p *< 0.001; *I*^2 ^= 0%; Figure 4).

### Safety outcome: incidence of adverse events

In either of the included studies, no severe adverse events associated with NPPV were reported.

### Publication bias

**Figure 5:**
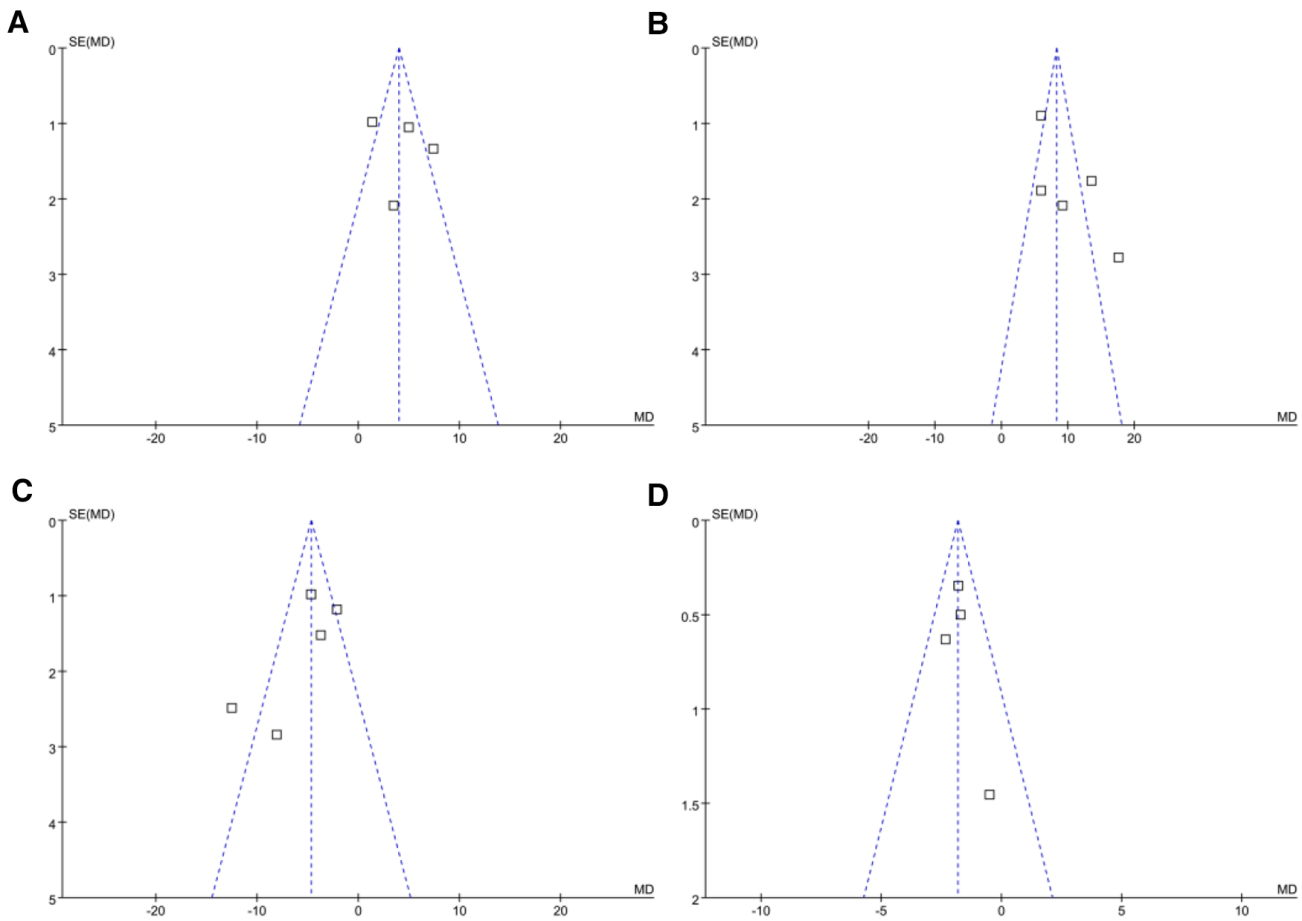
Funnel plots evaluating the publication biases of the meta-analyses. (**A**) Funnel plots for the meta-analysis of SaO_2_; (**B**) Funnel plots for the meta-analysis of PaO_2_; (**C**) Funnel plots for the meta-analysis of PaCO_2_; and (**D**) funnel plots for the meta-analysis of LOH.

The funnel plots for the meta-analyses comparing the effects of NPPV on SaO_2_, PaO_2_, PaCO_2_, and LOH are shown in Figures 5A–D. The symmetry of these plots indicates a low risk of publication bias. Egger's regression tests were not performed due to the limited studies included (four or five studies for each outcome). Only two or three studies were included, making it difficult to estimate the publication biases underlying the other results.

## Discussion

In this meta-analysis, we retrieved all available RCTs which evaluated the efficacy and safety of NPPV for children with acute asthma. The results showed that NPPV based on conventional therapy could significantly improve gas exchange within four hours after the initiation of the treatment, as evidenced by improved SaO_2_, increased PaO_2_, and decreased PaCO_2_ compared to conventional therapy alone. Moreover, the RR and asthma symptomatic scores were also acutely improved within four hours after the initiation of NPPV. The LOH was also significantly reduced for children who received NPPV. No severe adverse events related to NPPV were reported. These findings suggest that NPPV may be an effective and safe treatment strategy next to conventional treatment for pediatric patients with acute asthma.

To the best of our knowledge, there have not been many meta-analyses examining the function of NPPV in kids with acute asthma. In this meta-analysis, we retrieved relevant RCTs from five common English and Chinese electronic databases and summarized the current evidence regarding the efficacy and safety of NPPV for children with acute asthma. Because of the potential differences in the disease status and treatment tolerance of children and adults with acute asthma (4, 33), we carefully selected studies that included only pediatric patients. In addition, only RCTs were included, potentially minimizing the influences of confounding patients or study characteristics. Moreover, multiple outcomes were investigated, such as the acute changes of BGA parameters that reflect gas exchange, acute changes of RR and clinical symptomatic asthma scores, and the overall LOH, which all indicate a favorable role of NPPV. Finally, influencing analysis was performed, and the consistent results suggested the robustness of the finding, which was not primarily contributed by either of the included studies.

The potential benefits of NPPV for children with acute asthma may be multifactorial. As mentioned previously, using NPPV earlier in high-risk children with acute severe asthma could reduce their work of breathing, and more time could be given for the pharmacological treatment to reach maximum effect (34). This could be reflected by the acutely improved BGA parameters, reduced RR, and favorably changed symptomatic scores following NPPV in children with acute asthma in this meta-analysis. In recent large-scale retrospective studies involving both pediatric and adult patients with acute asthma exacerbation, the use of noninvasive ventilation was linked with a reduced likelihood of receiving invasive mechanical ventilation and a lower mortality rate in the hospital, suggesting that NPPV may be effective in improving the clinical outcomes (7). A recent survey with the Virtual Pediatric Systems database showed that from 2009 to 2019, a halving in the use of intubation has occurred in pediatric asthma, while a more than doubling in the use of noninvasive ventilation has occurred (35), which may also reflect the efficacy and tolerability of NPPV in real-world clinical practice. Interestingly, for pediatric outpatients with asthma, NPPV was demonstrated to reduce pulmonary inflammation and exercise-induced bronchospasm and increase asthma control and exercise durability (36, 37). To assess the long-term benefit of NPPV in children with asthma, large-scale RCTs are required.

In real-world clinical practice, determining when to initiate NPPV and which model of NPPV (BiPAP, PEP, or PEEP) to use in young children with respiratory disorders, including acute asthma, could be complicated. Since no evidence-based recommendation could be obtained as to the superiority of one model of NPPV over another in determining the initial treatment setting for NPPV, the medical team will consider hospital protocols, the availability of appropriate staff like respiratory therapists and nurses, and the degree of monitoring desired. More importantly, the choice and adjustment depend on the response and tolerability to the initial NPPV setting, jointly determined by the respiratory therapy, nursing, and medical teams. Overall, the meta-analysis results support the use of NPPV in pediatric patients with acute asthma. More studies and observations are needed to determine the optimal model and parameters of NPPV in this clinical circumstance.

On the other hand, besides NPPV, other non-invasive ventilation strategies have also shown satisfying efficacy and safety for children with acute asthma, such as HFNC (38). The mechanisms of HFNC involve delivering a high flow rate that exceeds inspiratory demand flow, providing minimal end-distending pressure, generating nasopharyngeal pressure, and reducing airway resistance (38). A pilot study showed that HFNC appears superior to conventional oxygen therapy for reducing respiratory distress within the first 2 h of treatment in children with moderate-to-severe asthma exacerbation refractory to first-line treatment (39). Another study in children with bronchiolitis suggested that FNC may be an effective and pleasant alternative to NPPV, showing similar efficacy in improving the respiratory rate, PaCO_2,_ and symptom score (40). HFNC could significantly elevate PaO2 and RR compared to conventional therapy, making it a promising option for patients with severe bronchial asthma complicated with respiratory failure (41). Interestingly, another retrospective analysis enrolling 42 children with severe acute asthma showed that HFNC is a lower level of respiratory support than NPPV, which could potentially delay the initiation of NPPV in these patients (42). Generally, HFNC is effective and well-tolerated in pediatric patients with acute asthma, which has been well-applied in real-world clinical practice as a midway point between low-flow oxygen devices and NPPV (43). Further studies are required to determine the optimal strategy and protocol for non-invasive ventilation in pediatric patients with acute asthma.

This study also has limitations. Firstly, although we aimed to pool the current available RCTs, the total number of studies and sample sizes for each meta-analysis outcome remained small. The results need to be validated by large-scale RCTs. Moreover, heterogeneity was significant among the included studies, which may be explained by differences in patient characteristics, concurrent therapy, the severity of the disease, and modes and settings of NPPV. However, we could not determine the source of heterogeneity since limited studies are available. Studies are warranted in the future to determine if the benefits of NPPV are consistent in studies with different ventilatory modes and settings. Furthermore, future clinical studies should investigate the effects of NPPV on the clinical outcomes of pediatric patients with acute asthma. Finally, further research will be needed to determine the best NPPV model for treating children with acute asthma exacerbations.

In conclusion, the results of the meta-analysis indicate that early use of NPPV in pediatric patients with acute asthma could acutely improve the BGA parameters of gas exchange and asthma symptoms and shorten the overall LOH without any severe adverse events related to the treatment. These findings support using NPPV as an adjuvant treatment to conventional therapies for children with acute asthma exacerbation.

## Data Availability

The original contributions presented in the study are included in the article further inquiries can be directed to the corresponding author/s.
